# Radiation induces ESCRT pathway dependent CD44v3^+^ extracellular vesicle production stimulating pro-tumor fibroblast activity in breast cancer

**DOI:** 10.3389/fonc.2022.913656

**Published:** 2022-08-29

**Authors:** Gene Chatman Clark, James David Hampton, Jennifer E. Koblinski, Bridget Quinn, Sitara Mahmoodi, Olga Metcalf, Chunqing Guo, Erica Peterson, Paul B. Fisher, Nicholas P. Farrell, Xiang-Yang Wang, Ross B. Mikkelsen

**Affiliations:** ^1^ Virginia Commonwealth University, Richmond, VA, United States; ^2^ Department of Biochemistry, Virginia Commonwealth University, Richmond, VA, United States; ^3^ Department of Pathology, Virginia Commonwealth University, Richmond, VA, United States; ^4^ Department of Radiation Oncology, Virginia Commonwealth University, Richmond, VA, United States; ^5^ University of Virginia, Charlottesville, VA, United States; ^6^ Department of Human Molecular Genetics, Virginia Commonwealth University, Richmond, VA, United States; ^7^ VCU Massey Cancer Center, Virginia Commonwealth University, Richmond, VA, United States; ^8^ Virginia Commonwealth University (VCU) Institute of Molecular Medicine, Virginia Commonwealth University, Richmond, VA, United States; ^9^ Department of Chemistry, Virginia Commonwealth University, Richmond, VA, United States

**Keywords:** cancer associated fibroblasts (CAF), cancer stem cell (CSC), extracurricular vesicles (EVs), radiotherapy, radioresistance, ESCRT pathway, CD44, heparan sulfate (HS)

## Abstract

Despite recent advances in radiotherapeutic strategies, acquired resistance remains a major obstacle, leading to tumor recurrence for many patients. Once thought to be a strictly cancer cell intrinsic property, it is becoming increasingly clear that treatment-resistance is driven in part by complex interactions between cancer cells and non-transformed cells of the tumor microenvironment. Herein, we report that radiotherapy induces the production of extracellular vesicles by breast cancer cells capable of stimulating tumor-supporting fibroblast activity, facilitating tumor survival and promoting cancer stem-like cell expansion. This pro-tumor activity was associated with fibroblast production of the paracrine signaling factor IL-6 and was dependent on the expression of the heparan sulfate proteoglycan CD44v3 on the vesicle surface. Enzymatic removal or pharmaceutical inhibition of its heparan sulfate side chains disrupted this tumor-fibroblast crosstalk. Additionally, we show that the radiation-induced production of CD44v3^+^ vesicles is effectively silenced by blocking the ESCRT pathway using a soluble pharmacological inhibitor of MDA-9/Syntenin/SDCBP PDZ1 domain activity, PDZ1i. This population of vesicles was also detected in the sera of human patients undergoing radiotherapy, therefore representing a potential biomarker for radiation therapy and providing an opportunity for clinical intervention to improve treatment outcomes.

## Introduction

Fibroblasts are versatile cells contributing to the structural integrity and wound healing responses of most tissues. As a critical component of the tumor microenvironment (TME), cancer associated fibroblasts (CAFs) support tumors by stimulating angiogenesis, metastasis, immunosuppression, and resistance to therapy (1). This is mediated by CAF secretion of dense extracellular matrix, expression of immunosuppressive surface proteins, and the production of paracrine signaling factors such as IL-6 which have been independently linked to treatment failure (2–6). For these reasons, CAFs have long been considered to be a tumor supporting population of stromal cells and their therapeutic modulation has been the focus of much effort (7). Evidence also suggests that CAF phenotypes might be plastic and responsive to complex signals present in the microenvironment, exhibiting both tumor supportive and tumor restrictive activity (8). Elucidating what signals determine the pro-tumor or anti-tumor function of fibroblasts in the TME may yield new therapeutic strategies.

Accumulating evidence suggests that tumor-derived extracellular vesicles (tEVs) play a critical role in the recruitment and education of CAFs (9). tEVs classically achieve these effects by the delivery of cancer cell-derived molecules such as noncoding RNA and proteins, capable of “reprogramming” fibroblasts to take on a cancer-associated phenotype (9). It has recently become clear that transmembrane proteins associated with the vesicular surface are also capable of eliciting robust signaling in target cells that leads to changes in cell character (10). One pathway thought to be responsible for the selective loading of transmembrane proteins onto tEVs is the Endosomal Sorting Complex Required for Transport (ESCRT) (11). Although an inhibitor of this vesicular production pathway has been reported previously, this has been disputed and may require clarification (12–14). Interestingly, it has been demonstrated that tEV production and characteristics can be altered by cellular stress such as that caused by chemotherapy and radiotherapy (15, 16). Other groups have investigated changes in the protein content and microRNA expression of IR-tEVs (17–19). It remains less well studied whether these stress-induced tEVs have differential effects on the function of stromal cells in the TME.

The part-time proteoglycan CD44 is detectable on the surface of tEVs from multiple cancer cell types where it has been reported to mediate the acquisition of cancer-associated phenotypes by mesenchymal cells (20, 21). In addition, this protein has been reported to be enriched in tEVs derived from breast cancer cells after exposure to DNA-damaging chemotherapy (22). Herein we demonstrate that ionizing radiation (IR) stimulates the production of heparan sulfate (HS)^+^CD44v3^+^ tEVs (IR-tEVs) from breast cancer cell lines and patients. These IR-tEVs in turn elicit enhanced pro-tumor activity of fibroblasts in the form of enhanced IL-6 production, induced radioresistance of breast cancer cells, and the expansion of a cancer stem-like cell (CSC) population. Furthermore, we show that the activity of IR-tEVs is dependent upon vesicular CD44v3 as well as its HS side chains and that their effects can be inhibited using pharmaceutical inhibitors of glycosaminoglycan function or by using a small molecule inhibitor of the MDA-9/Syntenin-1/SDCBP PDZ1 domain, PDZ1i (23–25) to block the ESCRT pathway.

## Materials and methods

### Antibodies and reagents

The following antibodies were used in this study: APC anti-human CD44v3 antibody (AB5088A, 1:300) was purchased from R&D Systems, (Minneapolis, MN). AF700 anti-mouse/human CD44 antibody (103025, 1:300), FITC anti-human CD81 antibody (cat. 349503, 1:300), APC anti-mouse/human CD44 Antibody (103012, 1:300), PE-Cy7 anti-human CD133 Antibody (393910, 1:300), and APC anti-mouse CD81 (104909, 1:300), and Zombie Aqua (423101), were purchased from Biolegend (San Diego, CA). Human FITC-Heparan Sulfate antibody (H1890-10, 1:300) was purchased from US Biological (Salem, MA). Human IL-6 ELISA kit and Mouse IL-6 ELISA kit were purchased from Biolegend (San Diego, CA).

Neutralizing anti-human IL-6 antibody (mabg-hil6-3) was purchased from *In vivo*gen (San Diego, CA). Heparinase I/III blend (H3917-50UN), chondroitinase ABC (C2905-2UN), and JSI-124 (C4493-1MG) were purchased from Sigma-Aldrich (Saint Louis, MO). PDZ1i was generously provided by Dr. Paul B. Fisher (23).

### Cell lines

E0771, LLC, and MRC5 cell lines were purchased from ATCC. All WT and genetically modified MDA-MB-231 and BT-549 cell lines were generously provided by the Koblinski lab (26, 27). MDA-MB-231 and E0771 CD44 KO cells were generated using the Synthego CRSPR KO kit version 2 according to the manufacturers protocol. DMEM (Gibco), RPMI (Gibco), PBS (Gibco), NEAA (Gibco), Pen/Strep (Gibco), and L-Glutamine (Gibco) were all purchased from ThermoFisher Scientific (Waltham, MA). FBS was purchased from R&D Systems (Minneapolis, MN). All cells were cultured with 5% CO_2_ at 37C. All cell lines were regularly tested for contamination with *Mycoplasma* using a PCR-based Mycoplasma Detection Kit (ATCC). Xylt1/2 KO cell lines were generously provided by the Koblinski and Farrell labs (28). Complete media refers to DMEM or RPMI supplemented with FBS Pen/Strep. Fibroblast media refers to complete RPMI supplemented with L-Glutamine.

### tEV isolation

Human breast cancer lines (MDA-MB-231, BT-549), mouse mammary cancer cell line E0771, and the mouse lung cancer cell line LLC were grown to 20% confluency. Human or mouse cells were then exposed to either a single dose of 4 or 10 Gy radiation respectively using the GammaCell Cesium Irradiator. After 48 hours, the media was replaced with fresh EV-depleted cDMEM (100k xg, 18 hours) and cells were incubated for 72 additional hours, until the cells approached 90% confluency. For isolation of tEV from untreated cells, cell lines were grown to 20% confluency and the media was changed to fresh DMEM and incubated for 72 hours, collected and centrifuged at 2000 xg for 10 minutes. After transfer to fresh 50 mL tubes, the media was centrifuged at 10k xg for 30 minutes, passed through a 0.22 um vacuum filter followed by ultracentrifugation at 100k xg for 3 hours. The resulting pellet was resuspended in 40 mL PBS and centrifuged again at 100k xg for 3 hours. The pellet was resuspended in 1 mL PBS and protein concentration was measured by Bradford assay.

### tEV preparation from tumor-bearing mice and patients

2 x 10^5^ E0771 cells were washed twice in PBS and injected subcutaneously into the flank of C57BL/6 mice aged 6-12 weeks from The Jackson Laboratory (Bar Harbor, ME). Once tumors had reached 1 cm in size, they were given a single dose of radiation using the Small Animal Radiation Research Platform (Xstrahl North America, Suwanee GA). Mice were sacrificed 5 days later and whole blood was collected into a tube containing EDTA to prevent clotting. Blood was centrifuged at 3000 xg for 25 minutes to obtain the serum. After dilution of 1 mL of serum with 9 mL PBS, tEVs were extracted as described above. All experiments were conducted in accordance with animal protocol AD20158 approved by VCU Institutional Animal Care and Use Committee.

tEVs were also extracted from deidentified serum samples from 6 consenting breast cancer patients of diverse histology and ER/PR/HER2 status in an institutional review board approved study, HM-12181. Blood was collected before the initiation of therapy and two weeks into treatment at 2 Gy daily doses of RT (20 Gy total). Serum (0.5 ml) was diluted in 9.5 mL PBS and tEVs were extracted as described above.

### Fibroblast stimulation

Whole mouse lungs from WT C57Bl/6 mice were minced and digested in FBS-free RPMI containing 1 mg/mL Collagenase IV (Sigma-Aldrich) and 20 µg/mL DNase I (Sigma-Aldrich) for 30 minutes at 37C with shaking. After incubation, the digested tissue fragments were passed through a sterile 70 um filter and centrifuged at 400 xg. The resulting pellet was resuspended and plated in 10 cm dishes in cRPMI supplemented with glutamine until the cells reached 90% confluency. Cells were then passaged and grown in 12 well plates until they reach 80% confluency, washed once in PBS, and the media replaced with fresh cRPMI containing 5 ug/mL cell line derived tEV or 50 ug/mL patient derived tEVs. 48 hours later, IL-6 was measured by ELISA and the resulting fibroblast conditioned media (FCM) was stored at -20C for future use in clonogenic assays.

### THP-1 stimulation

2 x 10^6^ THP-1 cells were plated in each well of a 6 well plate and incubated overnight in cRPMI containing 200 nM Phorbol 12-Myristate 13-Acetate (PMA). The cells were then washed with PBS and the media changed to fresh, cRPMI and the cells were allowed to settle for 24 hours before replacing media with fresh cRPMI containing 5 ug/mL MDA-MB-231 tEVs or IR-tEVs and conditioned media were collected 48 hours later.

### Mouse bone marrow derived macrophages

2 x 10^6^ mouse bone marrow cells were plated in each well of a 6 well plate and incubated in cDMEM supplemented with 20% L929 conditioned media and nonessential amino acids. The media was changed to fresh media on day 3 and day 5. On day 7, the cells were washed with PBS and the media was changed to cDMEM containing 5 ug/mL tEVs and conditioned media was collected after 48 hours.

### Cancer cell-fibroblast coculture

MDA-MB-231 cells were irradiated with 4 Gy using the GammaCell Cesium Irradiator. 1 x 10^4^ cancer cells were then incubated in a 12 well plate with 1 x 10^4^ MRC5 cells for 48 hours. Cells were washed once in PBS and the media was changed to fresh cRPMI with or without inhibitors. After 24 hours 100 uL of media was removed from the coculture and IL-6 was measured by ELISA. After 7 total days of coculture, CD44 and CD133 expression was measured by flow cytometry.

### Bead assisted flow cytometry

Fifty mg tEVs were incubated with 0.5 mL of 4 mm aldehyde/sulfate-latex beads (ThermoFisher, Waltham, MA) for 15 minutes at room temperature in a total volume of 50 ul before dilution with 1 mL PBS supplemented with 0.1% BSA and 0.01% NaN_3_ (BCB) and incubation overnight on rotation at 4°C for the purpose of blocking. Bead-coupled tEVs were then washed in BCB 2x to remove unbound material and then incubated with conjugated antibodies for 30 minutes at 4°C. Samples were washed in 1 mL BCB and resuspended in 250 mL BCB and analyzed with the BD LSRFortessa-X20 flow cytometer.

### Clonogenic assay

One hundred untreated or 2000 4-Gy-treated MDA-MB-231 cells were plated in 6 cm dishes. After 48 hours, the media was changed to 1/5 fibroblast conditioned media diluted in fresh cDMEM and allowed to incubate for another 72 hours. Culture media was changed to fresh cDMEM and the cells cultured for 1-2 weeks. Cells were then fixed in methanol and dyed with methylene blue. Data was reported as total number of counted colonies compared to non-irradiated, untreated controls (29).

### Blyscan assay

tEVs (50 mg) were stained with 1mL blyscan dye reagent for 30 minutes according to the manufacturers protocol (BioVendor R&D, Asheville, NC). The tEVs were diluted in 10 mL of PBS and centrifuged at 100k xg overnight. The resulting pellet was dried and resuspended in dissociation buffer and incubated overnight at 37°C. Absorbance was determined at 656 nM using a spectrophotometer.

### Statistical analysis

Statistical analysis was performed using GraphPad Prism 9. Data are expressed as mean ± SD. For assays comparing two groups, statistical significance was assessed using a student’s t-test. For assays comparing more than 2 groups, statistical significance was determined *via* ANOVA with multiple comparisons.

### Data availability

All data produced in this study was generated by the authors and is available upon request.

## Results and discussion

### Radiation stimulates tEV production leading to pro-tumor fibroblast activity

**Figure 1:**
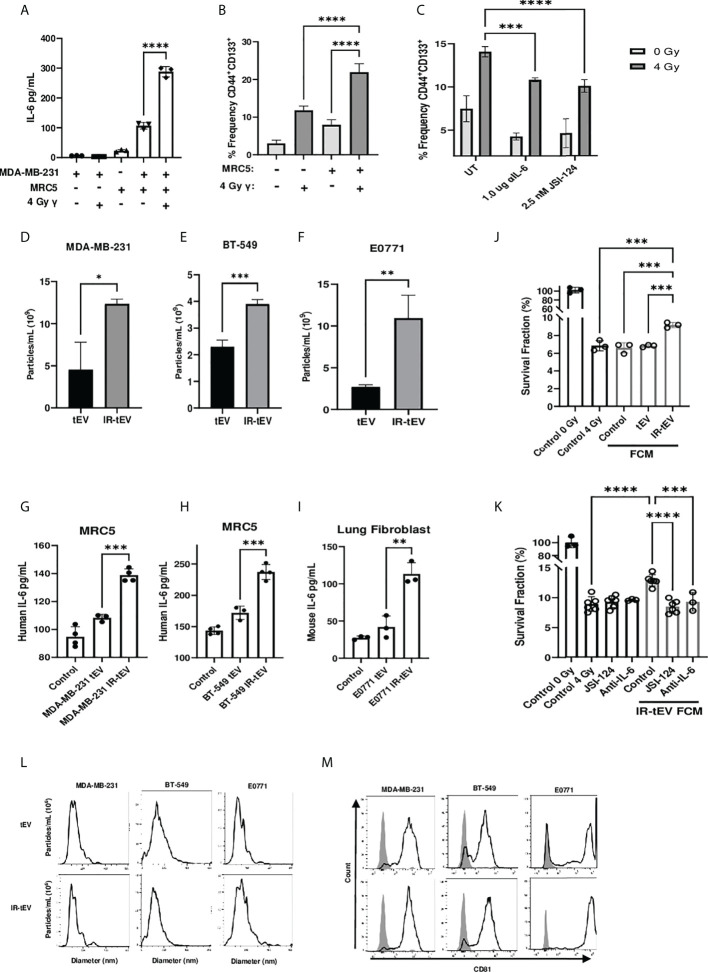
IR-tEV stimulate pro-tumor fibroblast activity. **(A)** MDA-MB-231 breast cancer cells were cocultured with fibroblasts. IL-6 expression was measured *via* ELISA **(A)** and CD44 and CD133 expression levels were quantified *via* flow cytometry **(B, C)** MDA-MB-231 cocultured with MRC5 cells with or without anti-IL-6 antibody or JSI-124. CD44 and CD133 expression were quantified *via* flow cytometry. Statistical significance for **(A-C)** assessed with ANOVA * = P < 0.05. **(D-F)** Human or mouse tEVs were collected from breast cancer cell conditioned media before or after exposure to 4 Gy or 10 Gy gamma radiation, respectively. Total particle number was assessed *via* particle tracking with the Zetaview Particle Tracker. **(G-I)** Fibroblasts were incubated with breast cancer cell line tEV or IR-tEVs as indicated. IL-6 levels were measured *via* ELISA. Statistical significance for **(D-I)** was assessed *via* students T test. * = P < 0.05. **(J)** Irradiated MDA-MB-231 were exposed to fresh media containing FCM described in **(G)** and radioresistance was assessed *via* clonogenic assay. X axis indicates which FCM from **(G)** was used in the incubation. **(K)** MDA-MB-231 were exposed to fresh media containing IR-tEV stimulated FCM described in **(G)**, in the presence or absence of STAT3 activation inhibitor or IL-6 neutralizing antibody, and radioresistance was assessed *via* clonogenic assay. Statistical significance for **(J, K)** was assessed with ANOVA * = P < 0.05. **(L)** The size distribution of tEVs collected from irradiated and untreated breast cancer cells was assessed *via* particle tracking with the Zetaview Particle Tracker. **(M)** Expression of the exosomal marker CD81 was assessed *via* bead assisted flow cytometry. ** denotes a P value < 0.005. *** denotes a P value < 0.0005. **** denotes a P value < 0.00005. These values were the results of ANOVA analyses performed on GraphPad.

*In vitro* coculture with fibroblasts has been demonstrated to enhance radioresistance in cancer cells of multiple origins. One possible reported mechanism is the activation of fibroblasts by cancer cell secreted factors and the subsequent production of fibroblast-derived paracrine signaling factors such as IL-6 (30). Whether this phenomenon can be stimulated by irradiation directly requires further investigation. To investigate whether breast cancer-fibroblast coculture induced IL-6 expression is enhanced by tumor irradiation, we cultured human fibroblasts with irradiated MDA-MB-231 cells and measured subsequent IL-6 production (**Figure 1A**). We found that irradiation of cancer cells immediately before coculture with fibroblasts did enhance IL-6 expression to a degree that could not be explained by induced IL-6 expression by the irradiated cells alone. This suggests that cancer cell irradiation stimulates the expression of a factor capable of activating fibroblasts within the TME.

CSCs are a radioresistant cancer cell population that may expand within tumors undergoing RT, a process thought to be facilitated by IL-6 expression derived from a potential fibroblast-CSC niche (31, 32). In order to demonstrate the biological relevance of the above finding, we tested whether IR-induced IL-6 expression from cocultured fibroblasts could induce the expansion of CSCs *in vitro*. Consistent with previous reports (33, 34), the frequency of CD44^+^CD133^+^ CSCs present in the coculture correlated with IL-6 expression (**Figure 1B**) and this phenomenon was greatly reduced in the presence of an IL-6 neutralizing antibody or pharmaceutical inhibition of STAT3 activation (**Figure 1C**). At the same time, blocking IL-6 in the coculture enhanced overall cell death by IR, indicating that IL-6 plays an active role in CSC expansion and the apparent CSC expansion is not simply an artifact of preferential killing of non-CSC cells under different conditions (**Supplemental Figure 1B**).

Cellular stress has been demonstrated to induce the expression of tEVs, in some cases mediating interactions with stromal cells that lead to enhanced cancer cell radioresistance (16). Considering this, we hypothesized that radiation may induce the secretion of tEVs capable of stimulating nearby fibroblasts, potentially explaining the enhanced fibroblast activity observed in our coculture. First, we examined the effect of IR on the production of tEVs. tEV were isolated from cancer-cell-line-conditioned media *via* ultracentrifugation and filtration before or after exposure to IR. Particle tracking analysis confirmed that IR stimulates the production of EVs by human and mouse mammary cancer cell lines MDA-MB-231, BT-549, and E0771 (**Figures s1D-F**). Mouse lung adenocarcinoma cells were also used to show that this phenomenon is not restricted to breast cancer cells (**Supplemental Figure 8A**). The size range of the particles collected before and after radiation was found to be comparable for these breast cancer line-derived tEVs (**Figure 1L**) as well as those collected from LLC (**Supplemental Figure 8C**). These particles were positive for the small vesicle marker CD81 (35) as assessed by bead assisted flow cytometry (**Figure 1M**, **Supplemental Figures 2E, 3F, 4D, 5E, 6G, 7E, 8D**).

EVs produced by tumor cells exposed to DNA damaging agents such as radiation and chemotherapy have been reported to stimulate cells of the immune system (36, 37). However, whether these therapy-induced vesicles are capable of influencing other stromal cells such as fibroblasts has not been explored. To test whether radiation-induced tEVs mediate the enhanced fibroblast stimulation observed in our coculture model, human and mouse fibroblasts were exposed to tEVs and IR-tEVs collected from breast cancer cells for 48 hours. Fibroblasts produced significantly more IL-6 when exposed to tEVs derived from irradiated breast cancer cells when compared to those from non-irradiated counterparts (**Figures 1G-I**). However, this was not seen when macrophages, another critical stromal cell type, were exposed to the same tEVs (**Supplemental Figures 1F-G**), indicating a cell specific effect. To further examine potential pro-tumoral fibroblast effects in the context of IR, we prepared fibroblast conditioned media (FCM) after their incubation with IR-tEV or non-IR tEV. Not surprisingly, exposure of breast cancer cells to IR tEV-stimulated FCM significantly enhanced post-radiation survival (**Figure 1J**), which was abrogated by both IL-6 neutralizing antibody and STAT3 inhibition (**Figure 1K**). The plating efficiency of these cells was not altered by the addition of the same FCM (**Supplemental Figures 1H-I**). These data suggest that fibroblast activation by IR-tEV represents a previously unreported mechanism for stimulation of the IL-6-STAT3 signaling pathway implicated in supporting tumor relapse and recovery from radiation damage (38, 39).

### The stimulatory activity of IR-tEV depends on vesicular CD44v3 expression

The activation of the cGAS-STING pathway has been implicated in stromal cell activation by RT, including in response to IR-tEVs (40, 41). However, using the soluble STING inhibitor H151 we found that STING plays little role in the response of breast cancer-fibroblast coculture to radiation (**Supplemental Figures 1C-E**). Preliminary unpublished studies in our laboratory suggested that IR-tEVs were associated with an increase in vesicular GAG content. This was intriguing because vesicular proteoglycans have been shown to be uniquely capable of mediating stimulation of mesenchymal cells by tEVs (42, 43). It has been previously observed that genotoxic agents can enhance the vesicular expression of the part-time proteoglycan CD44 on breast cancer derived tEVs (19, 22) where it has been demonstrated to mediate interaction with mesenchymal cells of the TME (20, 21). Considering our observation that IR enhances the expression of CD44 on the cell surface, the possibility that it might increase vesicular expression of CD44 was also examined. IR does indeed enhance expression of CD44 on the tEV surface (**Supplemental Figures 2G-I**).

**Figure 2:**
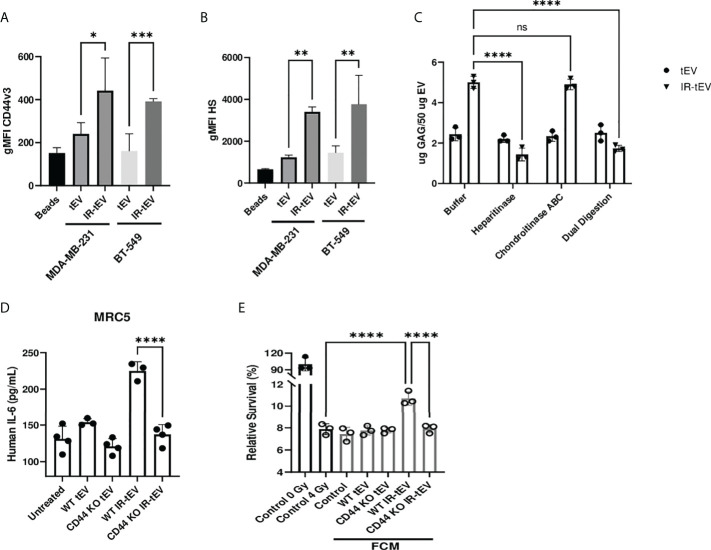
Radiation stimulates vesicular CD44v3 expression. **(A, B)** tEVs from breast cancer cells were assessed for CD44v3 **(A)** and HS **(B)** expression *via* flow cytometry. Control represents unbound beads incubated with antibody cocktail. **(C)** tEVs from MDA-MB-231 cells were treated overnight with either heparanase, chondroitinase ABC, or both. Gag levels were assessed *via* blyscan assay. Data points represent technical replicates. **(D)** MRC5 were incubated with tEVs collected from WT or CD44 KO MDA-MB-231. IL-6 levels were measured *via* ELISA. **(E)** MDA-MB-231 were exposed to fresh media containing FCM described in **(D)** and radioresistance was assessed *via* clonogenic assay. X axis indicates which fibroblast CM from **(D)** was used in the incubation. Statistical significance for **(A-E)** assessed with ANOVA * = P < 0.05. **(D, E)** Data points represent biological replicates. ** denotes a P value < 0.005. *** denotes a P value < 0.0005. **** denotes a P value < 0.00005. These values were the results of ANOVA analyses performed on GraphPad. “ns” represents a value for P that is greater than 0.05 and stands for “Not Significant”.

To explore and confirm this finding, we tested the possibility that the GAG-associated isoform of CD44, CD44v3, was present on IR-tEVs. Bead assisted flow cytometry demonstrated significantly increased CD44v3 protein levels on IR-tEVs compared to tEVs collected from the same cell lines without IR exposure (**Figure 2A**), suggesting that expression of this isoform may be stimulated directly by radiation. We were able to demonstrate that IR-tEVs from human breast cancer cell lines are indeed positive for HS (**Figure 2B**), a common substitution for CD44v3. Because CD44v3 can also be substituted with chondroitin sulfate, we incubated tEV derived from MDA-MB-231 with degradative enzymes specific for either HS or chondroitin sulfate ABC and then measured the resulting presence of GAG using a blyscan assay (**Figure 2C**). Because of the reported association between IR-tEVs and dsDNA, which can potentially bind to the blyscan dye, tEVs were also incubated with DNase I overnight and assessed for the presences of GAG (**Supplemental Figure 2B**). Incubation with heparanase abolished the surface presence of GAGs on IR-tEV while incubation with chondroitinase or DNase I had no effect. Because there is not a commercially available antibody specific for mouse HS, this strategy was also used to confirm the presences of HS on tEVs derived from irradiated E0771 cells (**Supplemental Figure 3B**) and LLC cells (**Supplemental Figure 8B**). Sulfated GAGs are linked to their protein cores *via* a short linker sequence ending in a Xylose (Xyl) residue *via* the activity of Xylosyl transferases 1 and 2 (44). We took advantage of Xylt1/2 KO MDA-MB-231 cell model to further validate the presence of GAG on IR-tEVs (**Supplemental Figure 2B**). To confirm that the presence of vesicular CD44 is necessary for HS expression on IR-tEVs, tEVs were collected from irradiated MDA-MB-231 (**Supplemental Figure 2B**) and E0771 CD44KO cells (**Supplemental Figure 3B**) and a blyscan assay was performed. In these cell lines, the absence of CD44 correlated with the absence of vesicular HS. To determine the biological significance of vesicular CD44 expression, MRC5 cells were incubated with IR-tEV isolated from WT or CD44 KO MDA-MB-231 cells. In the absence of CD44, IR-tEV lost the ability to stimulate fibroblasts, indicated by the loss of IL-6 expression by CD44KO IR-tEV stimulated fibroblasts compared to those stimulated by WT IR-tEVs (**Figure 2D**). As a result, FCM prepared following IR-tEV stimulation was no longer capable of enhancing the survival of irradiated MDA-MB-231 cells (**Figure 2E**).

**Figure 3:**
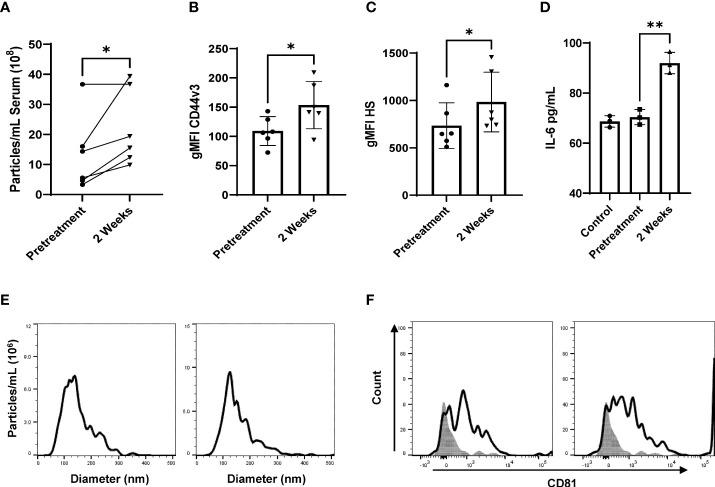
Radiation stimulates CD44v3^+^ IR-tEV circulation in breast cancer patients. **(A)** Total particle number of circulating breast cancer patient vesicles was assessed *via* particle tracking with the Zetaview Particle Tracker. **(B, C)** tEVs derived from breast cancer patient sera tEVs were bound to sulfoxide beads and HS and CD44v3 expression was measured *via* flow cytometry. Statistical significance for **(a-c)** was assessed with a paired T test. N=6. (d-h) Data points represent biological replicates. **(D)** MRC5 were incubated with tEVs collected and pooled from breast cancer patients. IL-6 levels were measured *via* ELISA Data points represent biological replicates. Statistical significance for was assessed with an unpaired T test. **(E)** The size distribution of tEVs collected from patients was assessed *via* particle tracking with the Zetaview Particle Tracker. **(F)** Expression of the exosomal marker CD81 was assessed *via* bead assisted flow cytometry. * denotes a P value < 0.05. These values were the results of ANOVA analyses performed on GraphPad.

To demonstrate that IR-tEVs accumulate in the bloodstream following RT, tEVs were isolated from the blood of breast cancer patients of multiple subtypes and histologies before and during RT and assessed for particle concentration and CD44v3 and HS expression. As shown in **Figure 3A**, an increase in the overall number of particles present in the sera of patients undergoing RT was observed. In addition, the fraction of particles displaying expression of CD44, CD44v3, and HS also increase significantly in patients during RT (**Supplemental Figure 2I**, **Figures 3B, C**). Finally, tEVs collected from patients during RT were more capable of stimulating MRC5 cell IL-6 expression than those isolated from the same patients before the start of therapy (**Figure 3D**). These particles were determined to fall within the appropriate EV size range and were positive for CD81 (**Figures 3E, F**). A mouse mammary cancer model was used to further validate this finding. WT and CD44KO E0771 cells were implanted into the flanks of C57BL/6 mice and the resulting tumors were given a single 10 Gy radiation dose using the Small Animal Radiation Research Platform (SARRP). Five days after radiation, serum was collected and circulating tEVs were isolated and assessed for particle number and the presence of GAG (**Supplemental Figures 4A, B**). We found that, similar to the human patient tEVs, the tEVs from irradiated tumor-bearing mice were clearly more abundant in the serum and positive for HS. However, this was not observed in mice bearing CD44 KO E0771 tumors. This finding was unexpected as irradiated CD44KO E0771 cells were observed to produce a similar level of particles compared to WT cells *in vitro* (**Supplemental Figure 3A**). We speculate that the change in surface charge that accompanies the loss of HS on the surface of these particles *in vivo* may alter their interactions with cell types known to filter tEVs from the blood such as myeloid cells in the lung and liver. Although radiation exposure of normal tissue was found to increase the overall number of circulating tEVs, these tEVs did not show enhanced expression of HS, suggesting that this phenomenon may be specific to cancer cells, although further analysis is needed.

### Secretion of CD44v3^+^ IR-tEVs is dependent upon ESCRT pathway components

**Figure 4:**
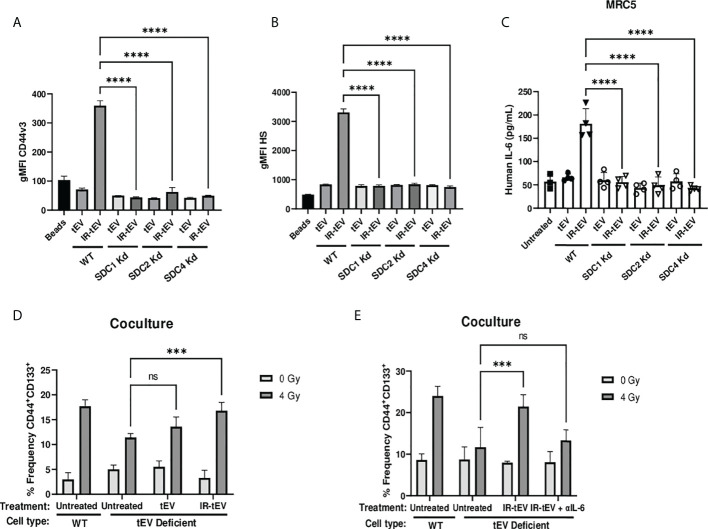
IR-tEV secretion is dependent upon the ESCRT pathway. **(A, B)** tEVs were collected from WT or SDC1, SDC2, or SDC4 KO MB-MDA-231 cells and CD44v3 **(A)** and HS **(B)** levels were assessed *via* bead assisted flow cytometry. X axis indicates from which parent cells the tEVs were derived. Control represents unbound beads incubated with antibody cocktail. **(C)** MRC5 cells were incubated with tEVs collected from WT or SDC1, SDC2, or SDC4 Kd MB-MDA-231 cells. IL-6 levels were measured *via* ELISA. **(D)** WT or SDC1 kd (tEV Deficient) MDA-MB-231 cells were cultured with fibroblasts with or without supplementation with WT tEVs. Protein surface markers were assessed *via* flow cytometry. **(E)** WT or SDC1 kd (tEV Deficient) MDA-MB-231 cells were cocultured with fibroblasts with supplementation of WT IR-tEVs in the presence of an IL-6 neutralizing antibody. Protein surface markers were assessed *via* flow cytometry. Statistical significance assessed with ANOVA P < 0.05. **(C-E)** Data points represent biological replicates. ** denotes a P value < 0.005. *** denotes a P value < 0.0005. **** denotes a P value < 0.00005. These values were the results of ANOVA analyses performed on GraphPad. “ns” represents a value for P that is greater than 0.05 and stands for “Not Significant”.

Radiation-induced tEV secretion has been suggested to be dependent upon the DNA damage-dependent activation of p53 and its gene product tumor suppressor-activated pathway 6 (TSAP6) (45). However, the mechanism of interaction between TSAP6 and tEV biogenesis machinery is unknown. The ESCRT pathway is one of the best described molecular mechanisms for intraluminal budding required for small vesicle biogenesis, involving the ordered assembly of multimeric complexes at the cytoplasmic surface of the endosome and the energy dependent budding and dissociation of intraluminal vesicles (11). This process relies on the binding of SDC proteoglycans to the PDZ1 domains of the scaffolding protein syntenin/MDA9 (11, 24, 25, 46–48). Using three different breast cancer models deficient for syndecan (SDC) protein function, we validated the necessity of the ESCRT pathway for the production of CD44v3^+^HS^+^ IR-tEVs (**Figures 4A, B**, **Supplemental Figures 6A-C, 7B**). SDC knockdown also clearly inhibited IR-induced stimulation of overall tEV production (**Supplemental Figures 5A, 6C, 7A**). Similar to CD44 KO, SDC knockdown also inhibited tEVs-stimulated IL-6 production by fibroblasts (**Figure 4C**) and these ESCRT deficient cells do not stimulate more IL-6 in coculture in response to radiation compared to untreated cells (**Supplemental Figure 5G**). Furthermore, the ability of these ESCRT deficient cells to stimulate CSC expansion in response to radiation was impaired (**Figure 4D**). This phenotype could be rescued by providing WT IR-tEVs in the culture media, but not in the presence of IL-6 neutralizing Ab (**Figure 4E**). These results suggest that IR-tEVs represent an exosomal phenomenon and that the ESCRT pathway may represent a viable pharmaceutical target for enhancing the response of breast cancer to RT.

**Figure 5:**
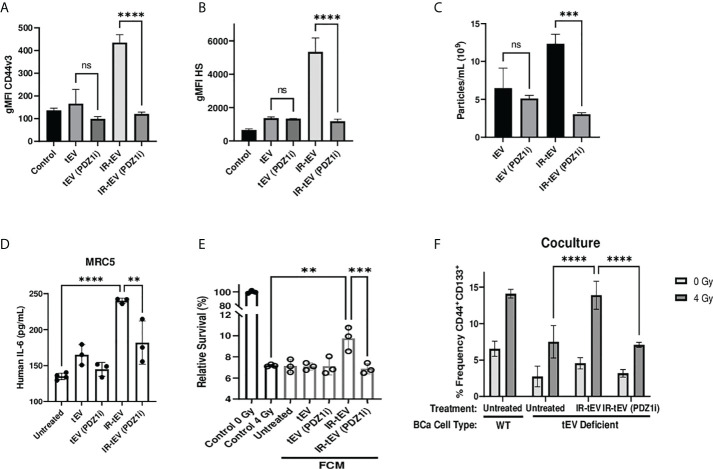
Pharmaceutical inhibition of the ESCRT pathway effectively silences IR-tEV activity. **(A-C)** tEVs were collected from untreated MB-MDA-231 cells or cell treated with 5 uM PDZ1i and CD44v3 **(A)** and HS **(B)** levels were assessed *via* bead assisted flow cytometry and overall tEV production **(C)** was assessed using the Zetaview Particle Tracker. **(D)** MRC5 cells were incubated with tEVs from PDZ1i treated MDA-MB-231 cells and IL-6 levels were measured *via* ELISA **(E)** MDA-MB-231 were exposed to fresh media containing FCM described in **(D)** and radioresistance was assessed *via* clonogenic assay. X axis indicates which FCM from **(D)** was used in each group. **(D-E)** Data points represent biological replicates. **(F)** WT or SDC1 Kd (tEV Deficient) MDA-MB-231 cells were cultured with MRC5 cells with or without the addition of WT tEVs. Protein surface markers were assessed *via* flow cytometry. ** denotes a P value < 0.005. *** denotes a P value < 0.0005. **** denotes a P value < 0.00005. These values were the results of ANOVA analyses performed on GraphPad. “ns” represents a value for P that is greater than 0.05 and stands for “Not Significant”.

Considering this, we tested whether pharmaceutical inhibition of this process mitigates the effects of IR-tEVs. Inhibition of ESCRT-dependent tEV production with the PDZ domain inhibitor PDZ1i (23, 25) caused the loss of both HS and CD44 expression in breast cancer cell derived IR-tEVs (**Figures 5A, B**, **Supplemental Figure 7B**) as well as overall IR-enhanced tEV production (**Figure 5C**, **Supplemental Figure 7A**). Similar to IR-tEVs collected from to SDC1 Kd cells, the IR-tEVs from PDZ1i treated cells were also incapable of simulating IL-6 expression from human fibroblasts (**Figure 5D**) or stimulating breast cancer cell radioresistance (**Figure 5E**). Finally, IR-tEVs collected from breast cancer cells exposed to PDZ1i failed to rescue SDC1 Kd CSC expansion in response to radiation in the co-culture model (**Figure 5F**). These results suggest that PDZ1i may represent a novel strategy for inhibiting ESCRT pathway-mediated IR-tEV production.

It is possible that the apparent enrichment of HS^+^CD44v3^+^ tEVs during RT is caused by proteoglycan shedding and subsequent contamination of the tEV preparation. In addition, contamination of the EV preparation with fragments of apoptotic bodies or plasma membrane-derived microvesicles could potentially cause the presence of HS and confound our investigation. However, neither proteoglycan shedding, nor apoptotic body or microvesicle formation has been reported to be dependent upon SDC family proteins or their binding partner syntenin as shown in the current study. This supports the conclusion that HS^+^CD44^+^ tEVs produced in the setting of radiation exposure are true exosomes, although further analysis is needed to confirm this.

### IR-tEV activity is dependent upon CD44v3 heparan sulfate side chains

**Figure 6:**
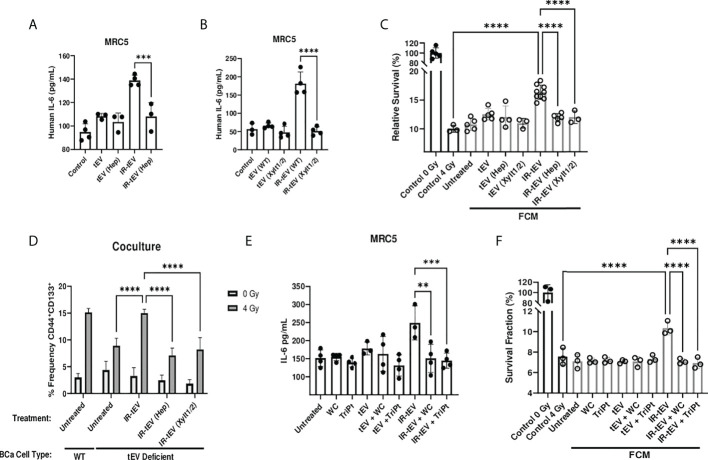
IR-tEV activity is dependent upon exosomal heparan sulfate. **(A)** MRC5 cells were stimulated with MDA-MB-231 derived tEVs enzymatically stripped of HS. IL-6 levels were measured *via* ELISA. **(B)** MRC5 cells were stimulated with tEVs collected from WT or Xylt1/2 KO MDA-MB-231 cells. IL-6 levels were measured *via* ELISA. **(C)** MDA-MB-231 were exposed to fresh media containing tEV stimulated MRC5 conditioned media described in **(A, B)** and radioresistance was assessed *via* clonogenic assay. X axis indicates which fibroblast CM from **(A, B)** was used in the incubation. **(D)** WT or SDC1 KO (tEV Deficient) cells were plated with MRC5 cells with or without the addition of WT IR-tEVs pretreated with heparanase. Protein surface markers were assessed *via* flow cytometry. **(E)** MRC5 cells were incubated with tEVs pretreated with 50 uM Triplatin or Werner’s Complex. IL-6 levels were measured *via* ELISA **(F)** MDA-MB-231 were exposed to fresh media containing IR-tEV stimulated MRC5 conditioned media described in **(E)** and radioresistance was assessed *via* clonogenic assay. X axis indicated what FCM from **(E)** was used in each incubation. Statistical significance assessed with ANOVA P < 0.05. Data points represent biological replicates. ** denotes a P value < 0.005. *** denotes a P value < 0.0005. **** denotes a P value < 0.00005. These values were the results of ANOVA analyses performed on GraphPad.

The activity of proteoglycans is often critically dependent on the presence of their associated GAGs. We next explored the possibility that HS present on the vesicular surface may be a viable target for neutralizing IR-tEV activity. First, we found that the ability of IR-tEV to stimulate IL-6 expression (**Figures 6A, B**) by fibroblasts as well as the ability of that FCM to stimulate post-radiation survival in breast cancer cells (**Figure 6C**) were silenced by either enzymatic removal or genetic ablation of HS. In addition, enzymatically treated IR-tEVs as well as IR-tEVs from Xylt1/2 KO failed to rescue ESCRT-dependent tEV deficient cells cocultured with fibroblasts (**Figure 6D**). The highly positively charged cobalt and platinum coordination compounds Werner’s Complex (WC) and Triplatin (TriPt) have been demonstrated to bind to HS, neutralizing its ability to interact with its molecular targets (28, 49). Due to the dependence of IR-tEV activity on the HS side chains attached to CD44v3, we also examined whether these drugs might be able to mitigate their effects. Preincubation of IR-tEV with either TriPt or WC inhibited their IL-6-stimulatory capacity (**Figure 6E**). In addition, both TriPt and WC inhibited the capacity of IR-tEV stimulated FCM to enhance breast cancer radioresistance (**Figure 6F**). These results confirm that, similar to other proteoglycans, the activity of tEV-associated CD44v3 is critically dependent upon its heparan sulfate side chains.

### Conclusions and future directions

A major factor limiting the success of RT is acquired radioresistance by surviving tumor cells, a process in which stromal fibroblasts or CAF have been heavily implicated (1, 50). However, recent clinical trials aimed at CAF ablation prior to or during therapy have failed to yield a clinical benefit, in some cases actually accelerating tumor progression (1). It has been suggested that this may reflect the underappreciated but critical tumor-restrictive role of certain CAF subpopulations present in the TME (8). New strategies that limit the tumor supportive functions of fibroblasts without limiting their tumor-restrictive capacity may allow for greater radio-sensitization of tumors. Herein, we report that RT stimulates the production of a distinct population of CD44v3^+^ tEVs by breast cancer cells which are capable of directly stimulating tumor-supportive fibroblast activity, resulting in enhanced cancer cell radioresistance and an expansion of breast CSCs. We also confirm the presence of these tEVs in the circulation of breast cancer patients during RT, suggesting an opportunity for clinical intervention. Additionally, we have provided two different strategies by which this might be achieved- *via* the inhibition of tEV-associated HS activity or inhibition of ESCRT pathway-mediated vesicle production. These findings represent an underappreciated pathway by which breast cancer develops radioresistance as well as a novel avenue by which the pro-tumor activity of CAFs can be restrained during RT.

The role that GAGs play in tumorigenesis and the tumor response to therapy has begun to receive significant attention due to the recent recognition of their impact on the character and development of the TME (51). Accordingly, anti-cancer therapeutics with GAG binding properties are currently in development. Our data suggests that IR-tEV activity is dependent upon the vesicular transmembrane proteoglycan CD44v3 and its associated HS side chains. Furthermore, we have shown that IR-tEV activity is neutralized by two drugs with GAG-neutralizing properties- TriPt and WC, thereby limiting breast cancer-fibroblast interplay during RT. This indicates a novel therapeutic strategy for enhancing the response of breast cancer to RT. As TriPt has also been demonstrated to be an effective cytotoxic chemotherapeutic (28), future studies are warranted to determine if concomitant therapy with TriPt and RT may produce a synergistic effect.

The molecular mechanisms of how HS or CD44 mediate the effects of IR-tEV on fibroblasts were not addressed by our study. Given the diverse interactions and binding partners reported for these molecules, many possibilities exist and elucidating this mechanism represents an important direction for future research. One critical function of HS is derived from its ability to simultaneously bind soluble and membrane associated growth factors and their tyrosine kinase receptors, stabilizing the resulting ternary complexes (52). One possible explanation for the role played by HS on the surface of IR-tEVs is that they initiate or enhance stimulation of fibroblasts by binding to an as yet unknown growth factor or cytokine, a mechanism common to GAG interactions with this cell type (53–55). Alternatively, it is possible that the HS associated with IR-tEVs stimulates fibroblasts more directly. GAG sulfation, length, and epimerization patterns are cell-and-tissue-specific and have been demonstrated to change with stress, in some cases leading to a gain of function (52). Finally, others have demonstrated that tEVs are capable of delivering functionally active CD44 to the mesenchymal cell membrane, resulting in an activated, pro-tumor phenotype (21, 56). It is possible that vesicular delivery of functional CD44v3 to fibroblasts by IR-tEVs could also explain our observations.

Using multiple human and mouse models, we have shown that the production of HS^+^CD44v3^+^ IR-tEVs is dependent upon SDC family proteins and that their activity can be pharmaceutically targeted using a soluble inhibitor of the MDA-9/Syntenin-1/SDCBP PDZ1 domain activity, PDZ1i (23). We would like to note that the utility of this approach may not be limited to inhibiting the activity of CD44v3^+^ IR-tEVs, as other vesicular components such as CD81 have also been implicated in mediating resistance to therapy (57). To our knowledge, this is the first report of a therapeutic antagonist of ESCRT-dependent tEV production targeting this protein interaction. The SDC-syntenin complex has also been demonstrated to play a role in the selective loading of proteins into the vesicular membrane (11). Thus, a more nuanced interaction between these proteins and CD44v3 than we have illustrated in this study is possible. For example, syntenin has been reported to interact with ubiquitinated membrane proteins, representing a potential mechanism by which SDC-syntenin complexes might select cargo for incorporation into EVs (58, 59). In addition, the cytoplasmic domains of SDC proteins represent the dominant form of bait tethering syntenin to the plasma membrane and are necessary for the interactions of its PDZ domains with alternate cargo (11). Therefore, we do not rule out the possibility that CD44 recruitment and delivery to the vesicular membrane could occur *via* a mechanism that involves ubiquitination of CD44 and subsequent binding by syntenin. Such a mechanism would not by itself, however, explain the loss of overall IR-tEV production caused by SDC KO or treatment with PDZ1i observed in our study. Further studies that include inhibition of alternative components of this pathway, such as inhibitors of PIP2 (60, 61) or the small GTPases RAB7 (62) and ARF6 (63), might help further elucidate this mechanism.

In summary, the targeting of IR-tEVs or their effects represents a novel opportunity to limit the recruitment of pro-tumor CAF activity stimulated by RT. We have demonstrated that this is feasible using two different approaches, both by inhibition of ESCRT pathway mediated vesicle production as well as neutralization of HS activity. In addition, based on our data, we speculate that other strategies could also be useful if given concurrently with RT, such as the use of anti-CD44 or anti-IL-6 neutralizing antibodies. Given the unique property of these IR-tEVs, additional studies of their effects on the TME, especially the behavior of other cell types such as immune cells are warranted.

## Data availability statement

The raw data supporting the conclusions of this article will be made available by the authors, without undue reservation.

## Author contributions

GC was responsible for designing and executing experiments, data interpretation, writing the manuscript, and generating hypotheses. JH and EP were responsible for designing and executing experiments, interpreting data, and generating hypotheses. SM and OM were responsible for executing experiments and interpreting data. JK, BQ, and NF were responsible for interpreting data, generating hypotheses, designing experiments, and supplying materials. PBF was responsible for interpreting data, supplying materials, and editing. X-YW and RM were responsible for interpreting data, generating hypotheses, designing experiments, editing, funding support, and supplying materials. All authors contributed to the article and approved the submitted version.

## Funding

The study was supported in part by Ruth L. Kirschstein Individual Predoctoral NRSA for MD/PhD and other Dual Degree Fellowships (1F30CA239564), National Institute of Health grants (CA175033, AI133595), VCU Massey Cancer Center Research Development Funds, and National Cancer Institute Cancer Center Support Grant to VCU Massey Cancer Center P30CA16059.

## Acknowledgments

We would like to thank Dr. Wenjie Liu, Dr. Jinyang Cai, and Dr. Zheng Liu for their wonderful advice and support.

## Conflict of interest

PBF is a scientific co-founder and has equity in InterLeukin Combinatorial Therapies, Inc. (ILCT). VCU also has equity in ILCT.

The remaining authors declare that the research was conducted in the absence of any commercial or financial relationships that could be construed as a potential conflict of interest

## Publisher’s note

All claims expressed in this article are solely those of the authors and do not necessarily represent those of their affiliated organizations, or those of the publisher, the editors and the reviewers. Any product that may be evaluated in this article, or claim that may be made by its manufacturer, is not guaranteed or endorsed by the publisher.
