# Supplementary material for: Radiation induces ESCRT pathway dependent CD44v3+ extracellular vesicle production stimulating pro-tumor fibroblast activity in breast cancer
Source: Front Oncol. 2022 Aug 29;12:913656. doi: 10.3389/fonc.2022.913656 (PMC9465418; doi:10.3389/fonc.2022.913656)
Supplement: Supplementary file 1 [file Presentation_1.pptx]

## Slide 1
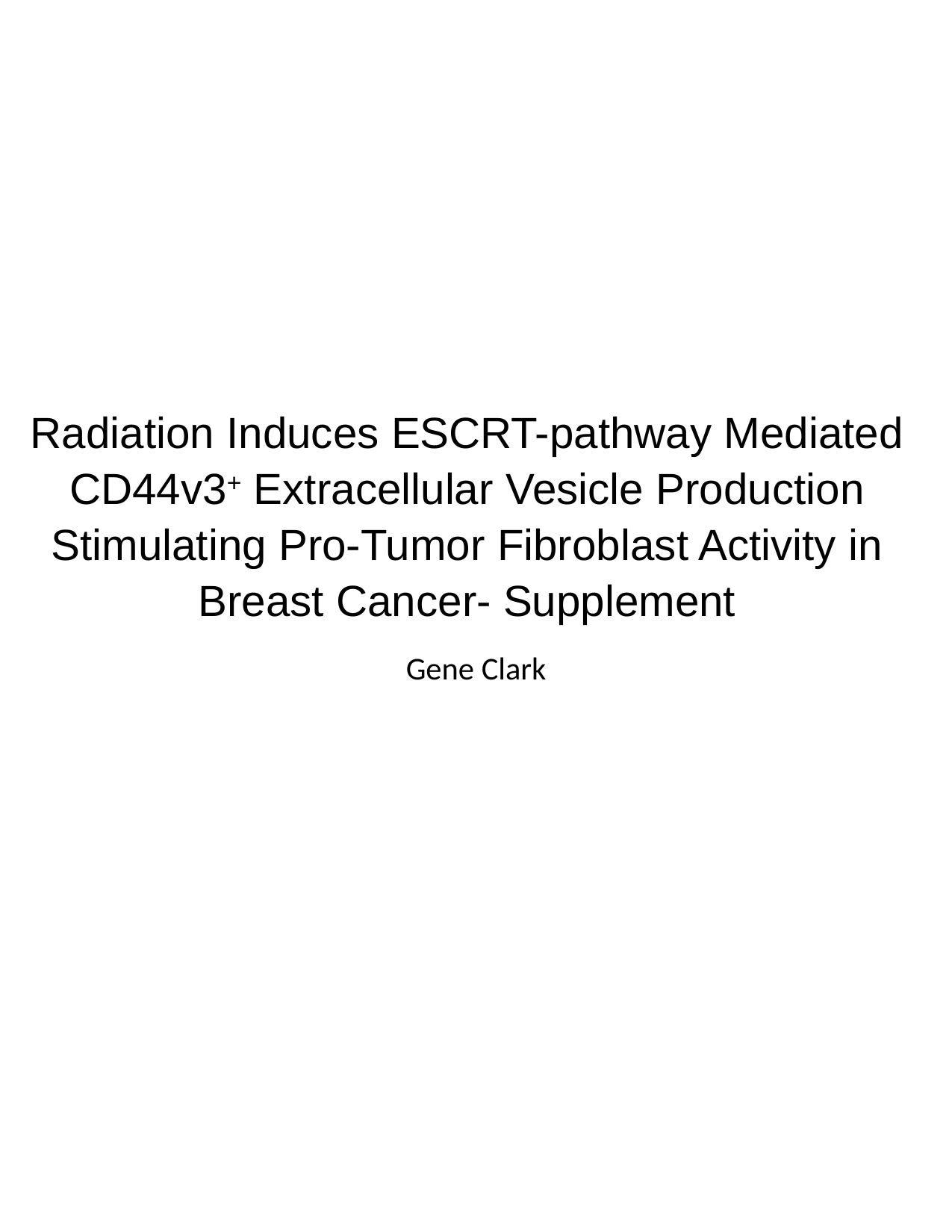

# Radiation Induces ESCRT-pathway Mediated CD44v3+ Extracellular Vesicle Production Stimulating Pro-Tumor Fibroblast Activity in Breast Cancer- Supplement
Gene Clark

## Slide 2
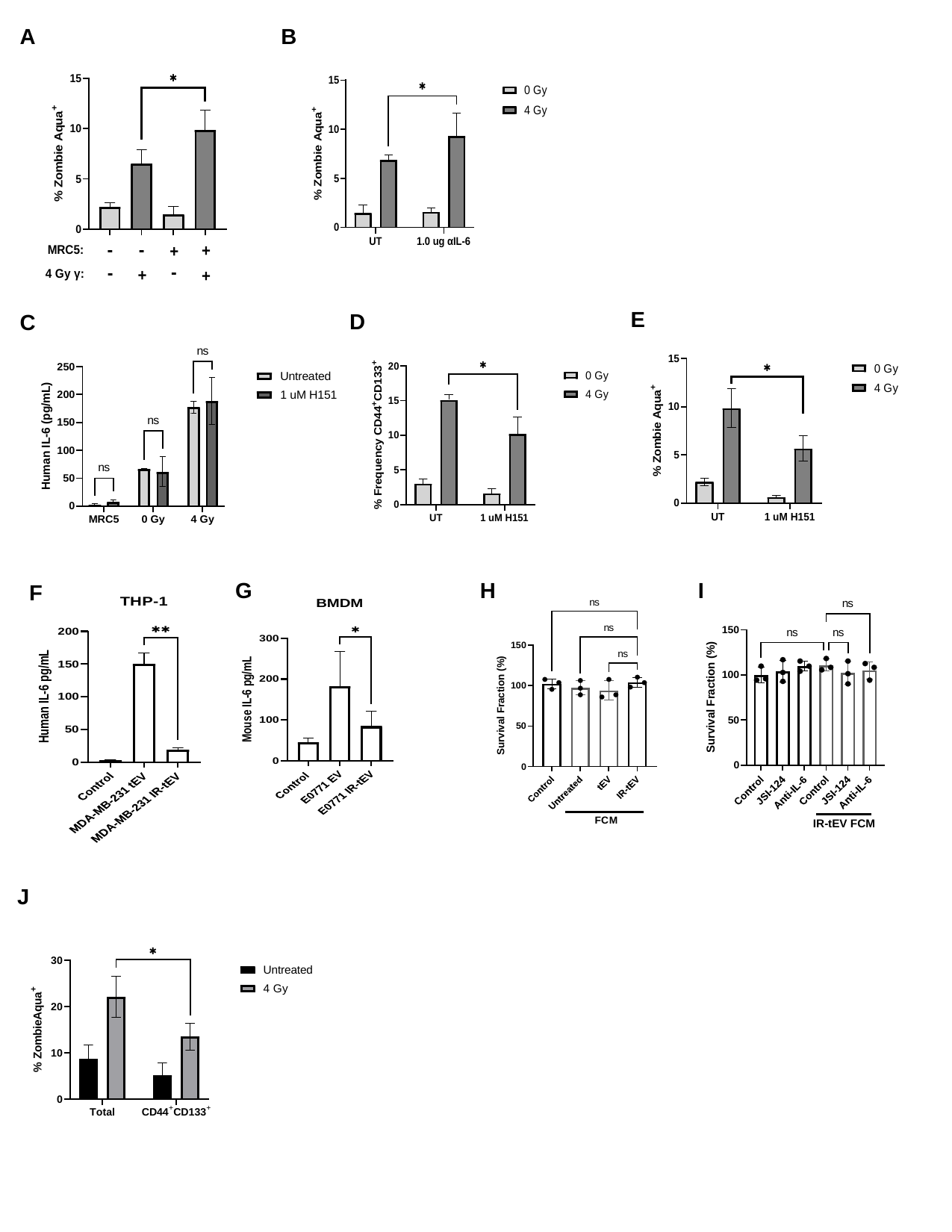

A
B
E
D
C
G
I
H
F
J

## Slide 3
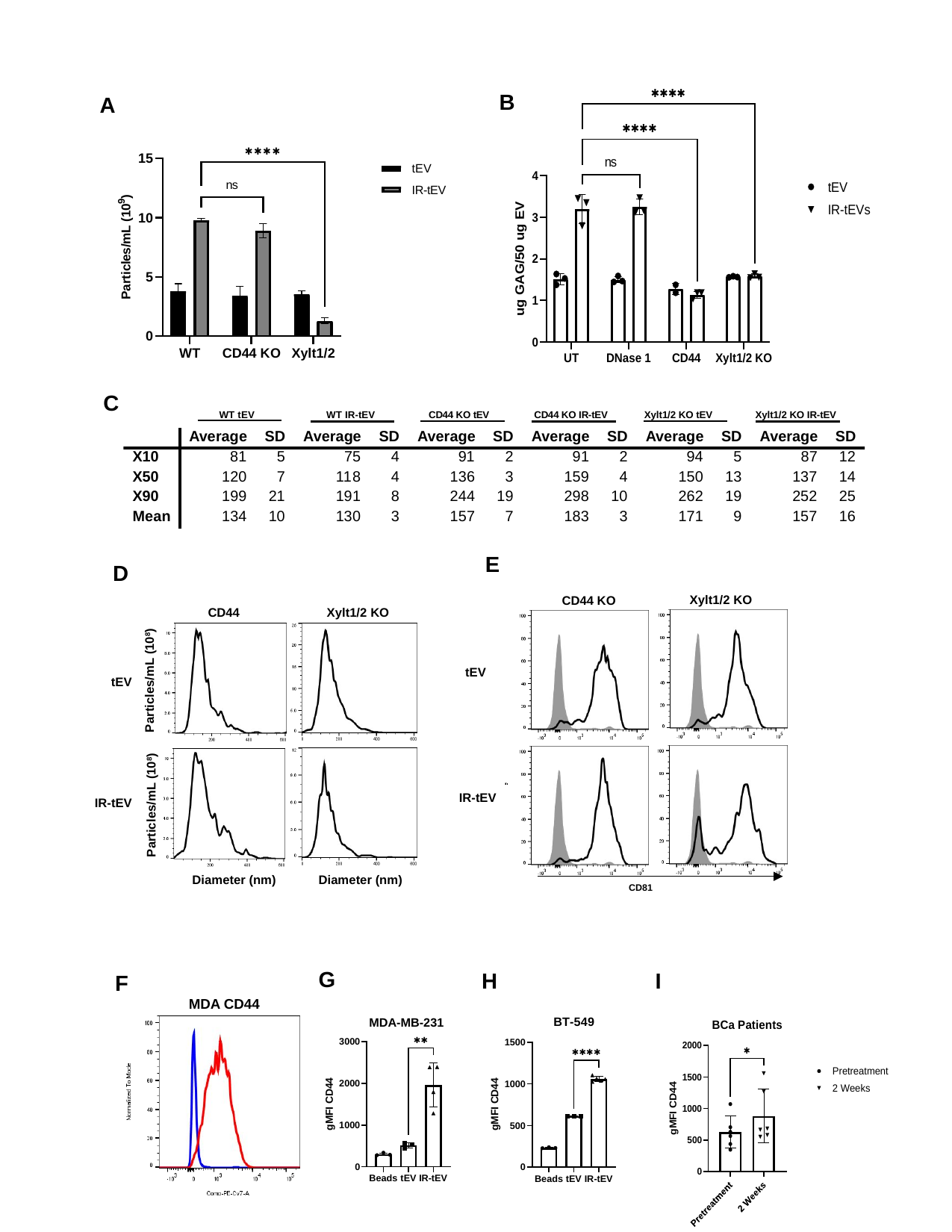

B
A
C
E
tEV
IR-tEV
 CD81
Xylt1/2 KO
CD44 KO
D
Xylt1/2 KO
CD44 KO
tEV
IR-tEV
Diameter (nm)
Diameter (nm)
Particles/mL (108)
Particles/mL (108)
G
I
H
F
MDA CD44

## Slide 4
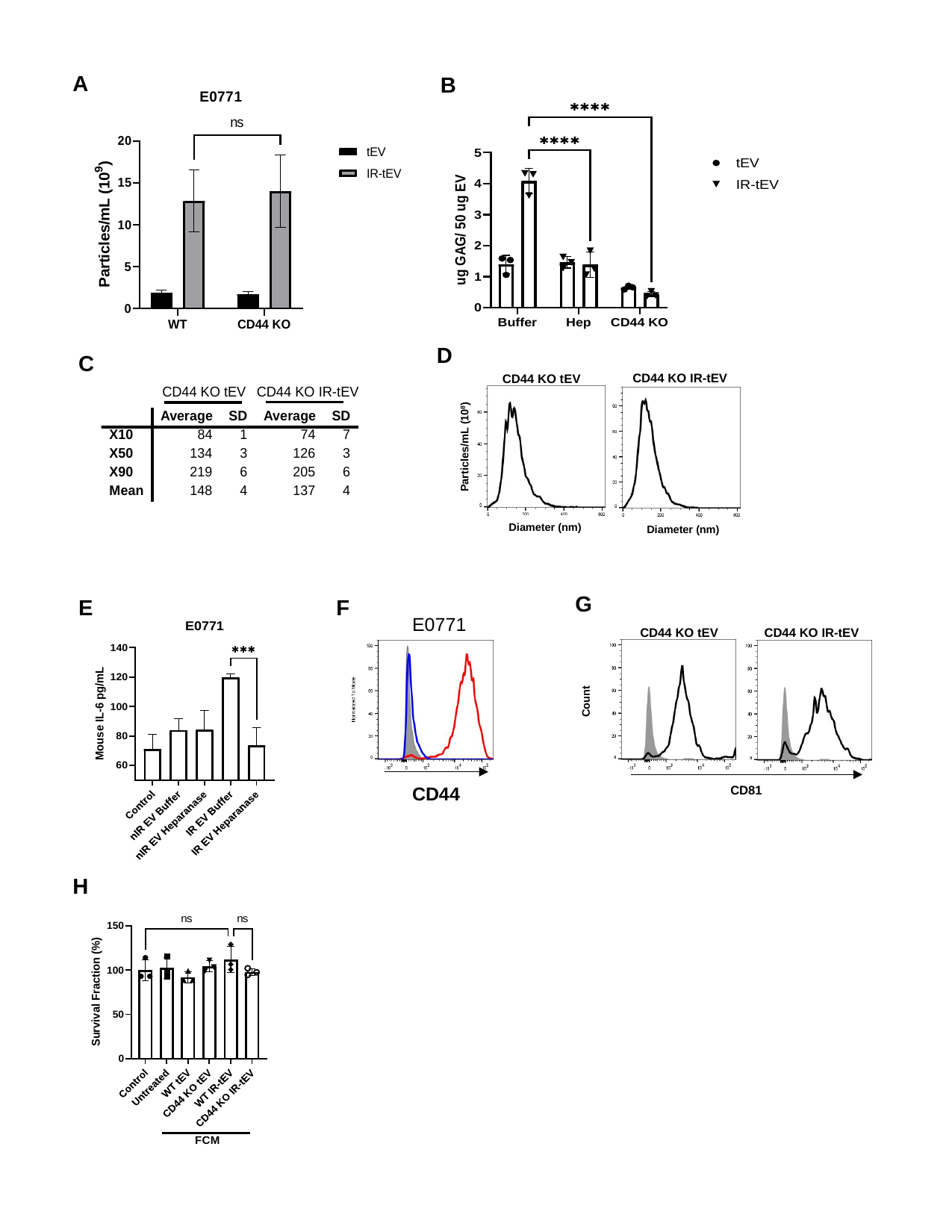

A
C
B
D
CD44 KO IR-tEV
CD44 KO tEV
Particles/mL (108)
Diameter (nm)
Diameter (nm)
G
CD81
CD44 KO tEV
CD44 KO IR-tEV
Count
E
F
E0771
CD44
H

## Slide 5
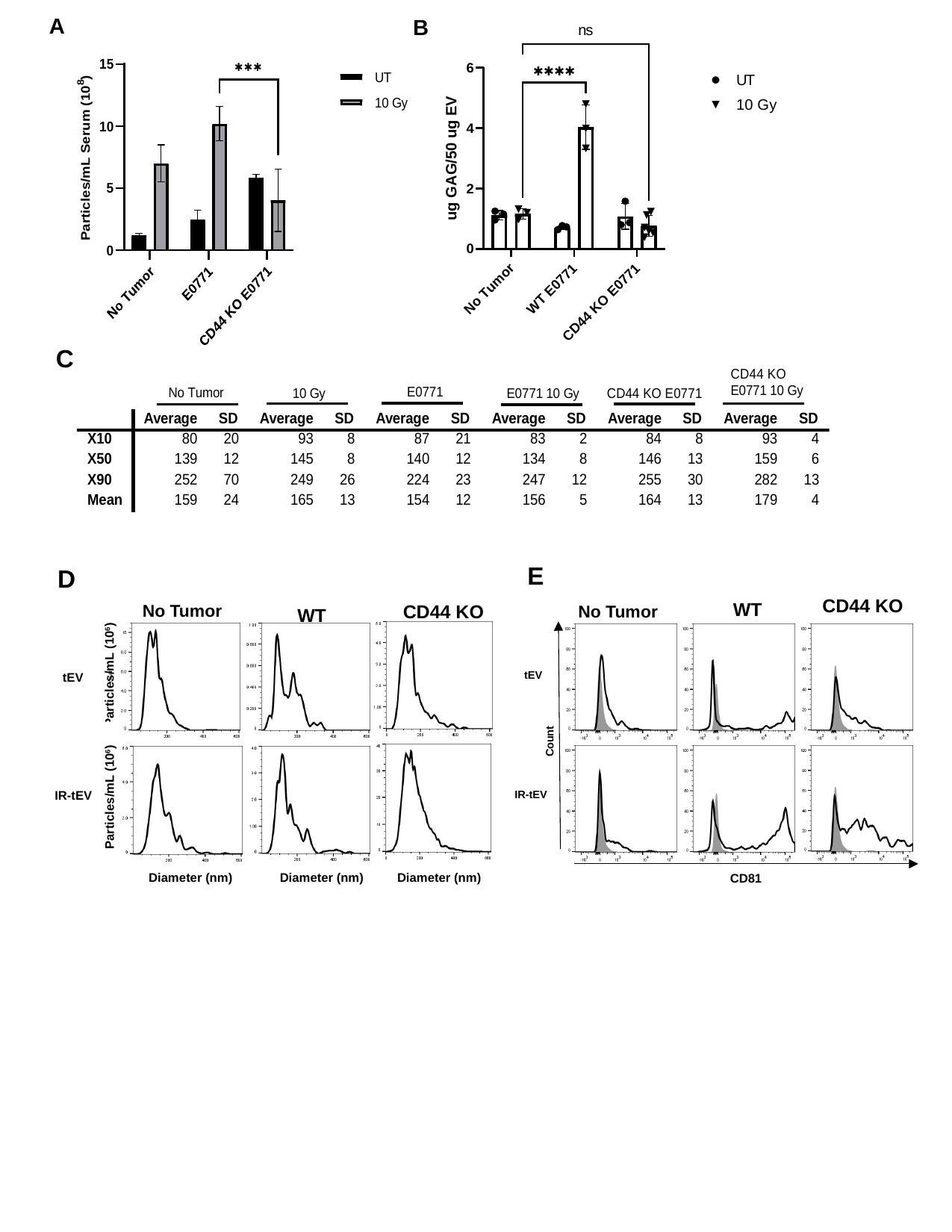

A
B
C
No Tumor
CD44 KO
WT
Particles/mL (106)
Diameter (nm)
tEV
IR-tEV
Diameter (nm)
Diameter (nm)
Particles/mL (106)
D
E
CD81
CD44 KO
WT
No Tumor
tEV
Count
IR-tEV

## Slide 6
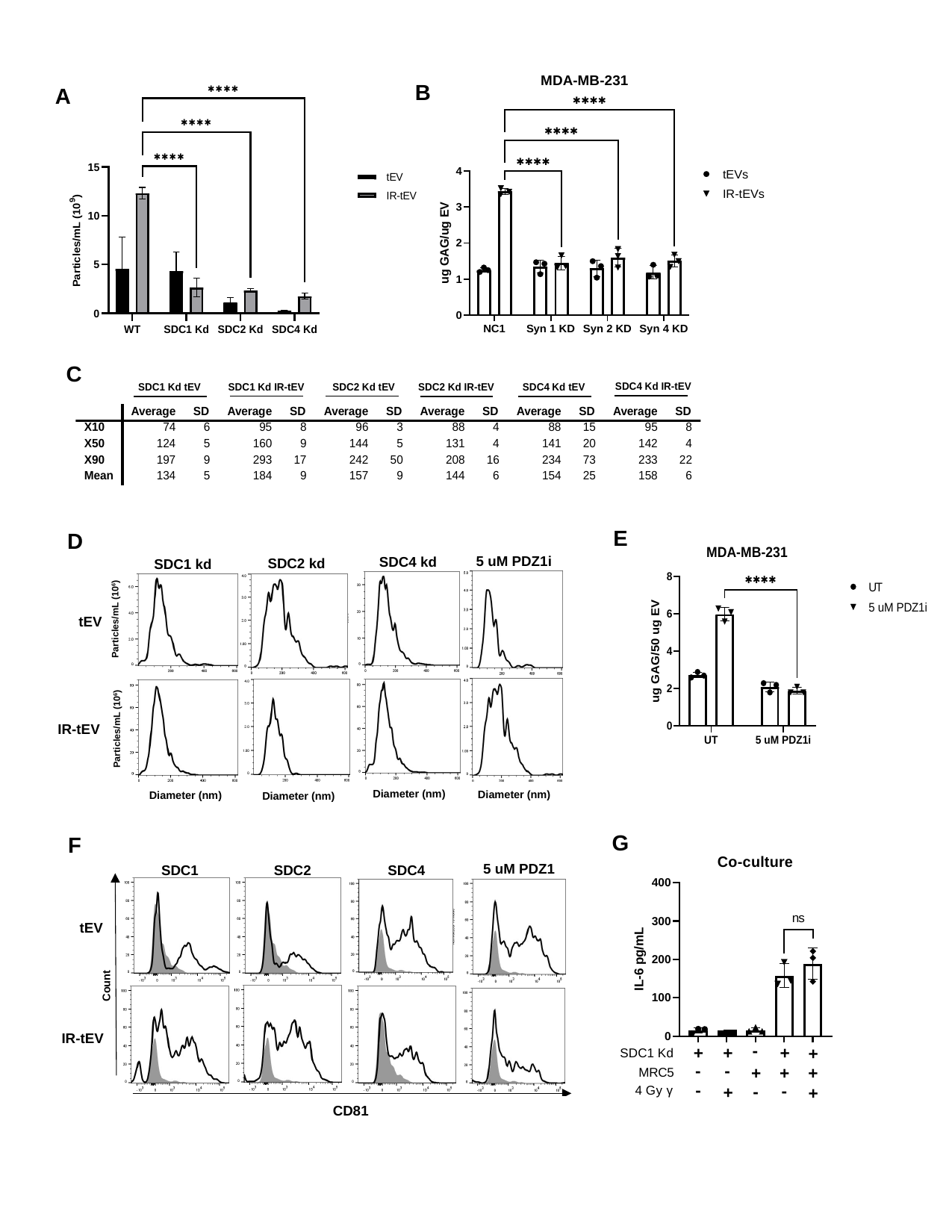

B
A
C
E
D
SDC4 kd
SDC2 kd
SDC1 kd
Particles/mL (106)
Particles/mL (106)
tEV
IR-tEV
5 uM PDZ1i
Diameter (nm)
Diameter (nm)
Diameter (nm)
Diameter (nm)
G
F
SDC2 kd
SDC4 kd
SDC1 kd
 CD81
tEV
IR-tEV
5 uM PDZ1
Count

## Slide 7
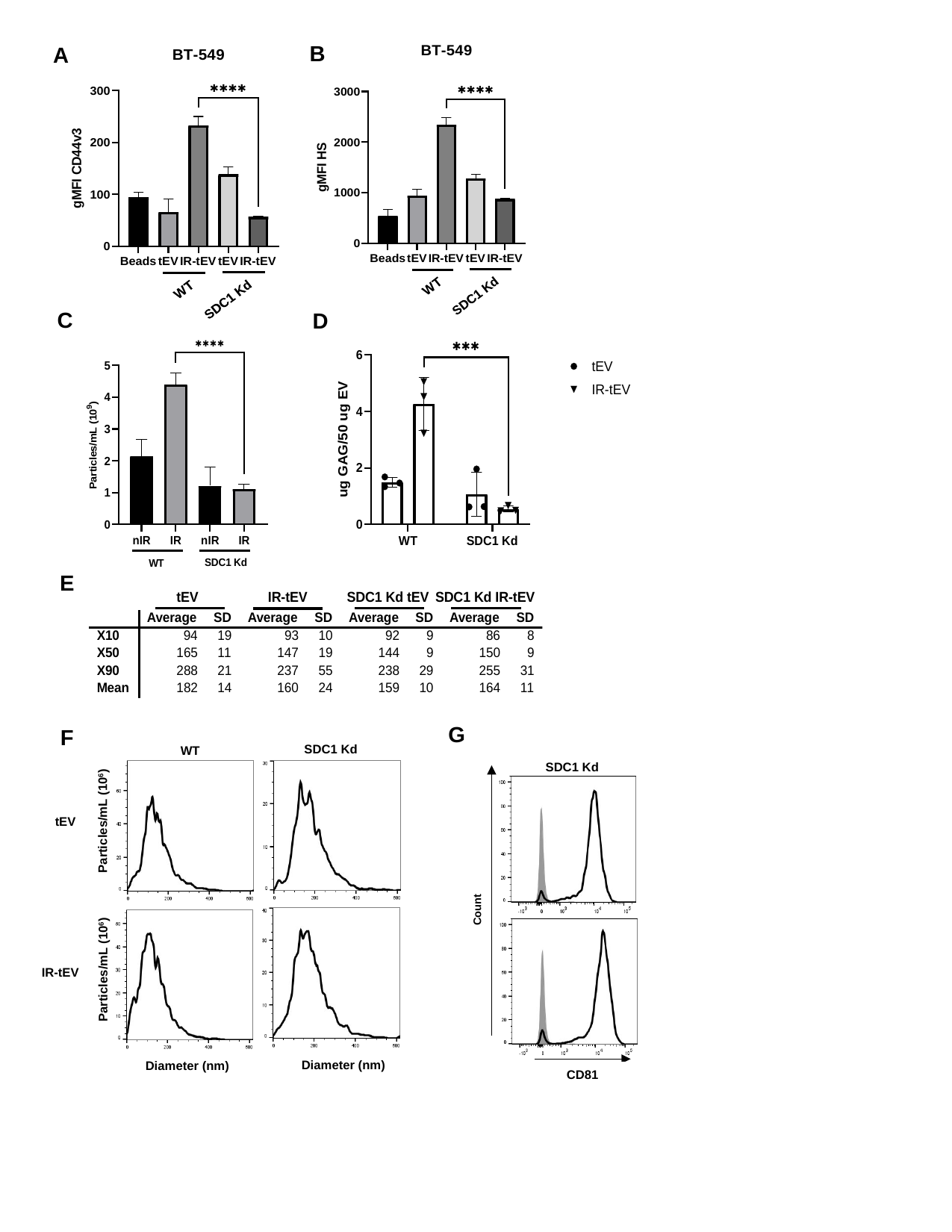

B
A
C
D
E
G
SDC1 Kd
CD81
Count
F
SDC1 Kd
WT
Particles/mL (106)
tEV
IR-tEV
Diameter (nm)
Diameter (nm)
Particles/mL (106)

## Slide 8
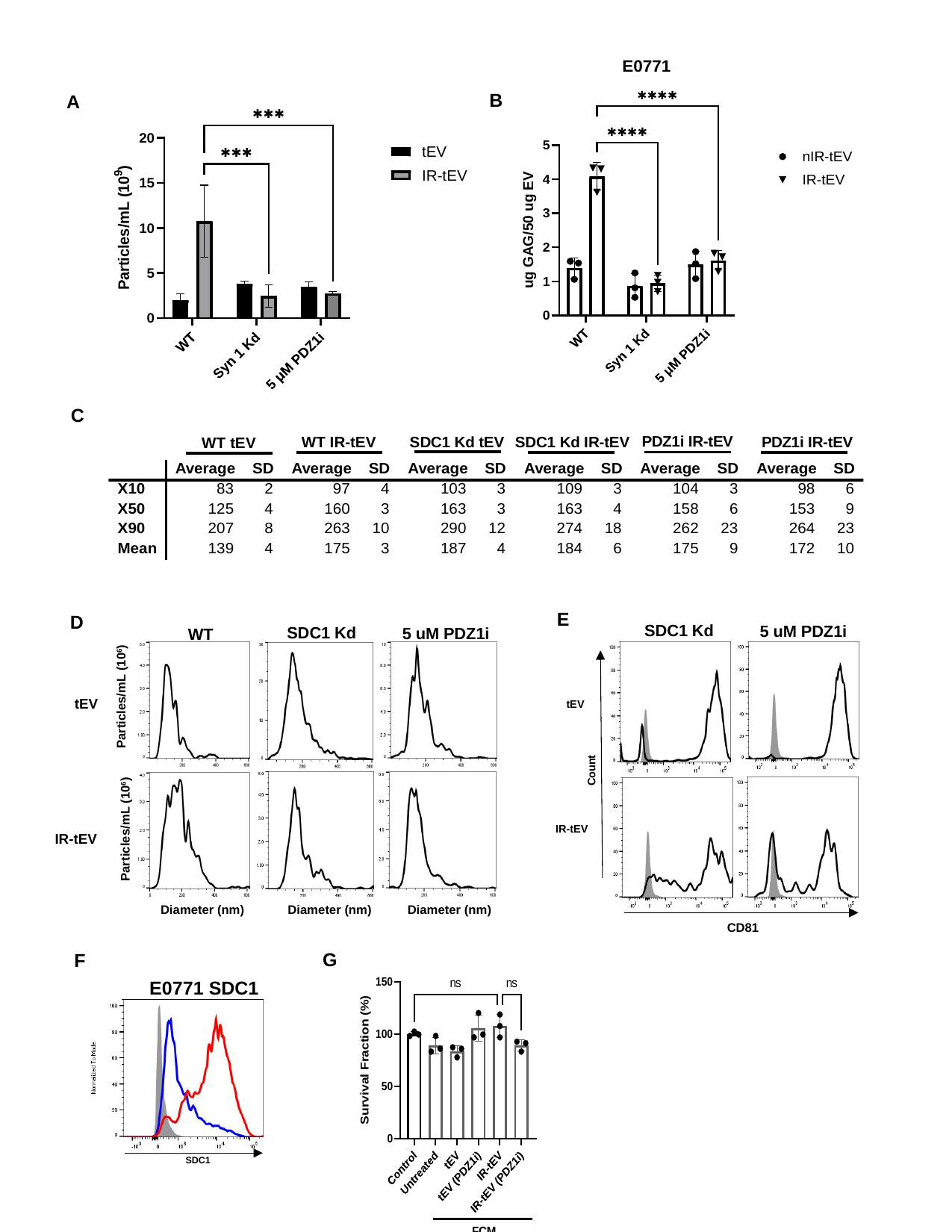

B
A
C
tEV
Particles/mL (106)
WT
IR-tEV
Diameter (nm)
Particles/mL (106)
Diameter (nm)
Diameter (nm)
SDC1 Kd
5 uM PDZ1i
D
E
SDC1 Kd
5 uM PDZ1i
CD81
tEV
Count
IR-tEV
G
F
E0771 SDC1
SDC1

## Slide 9
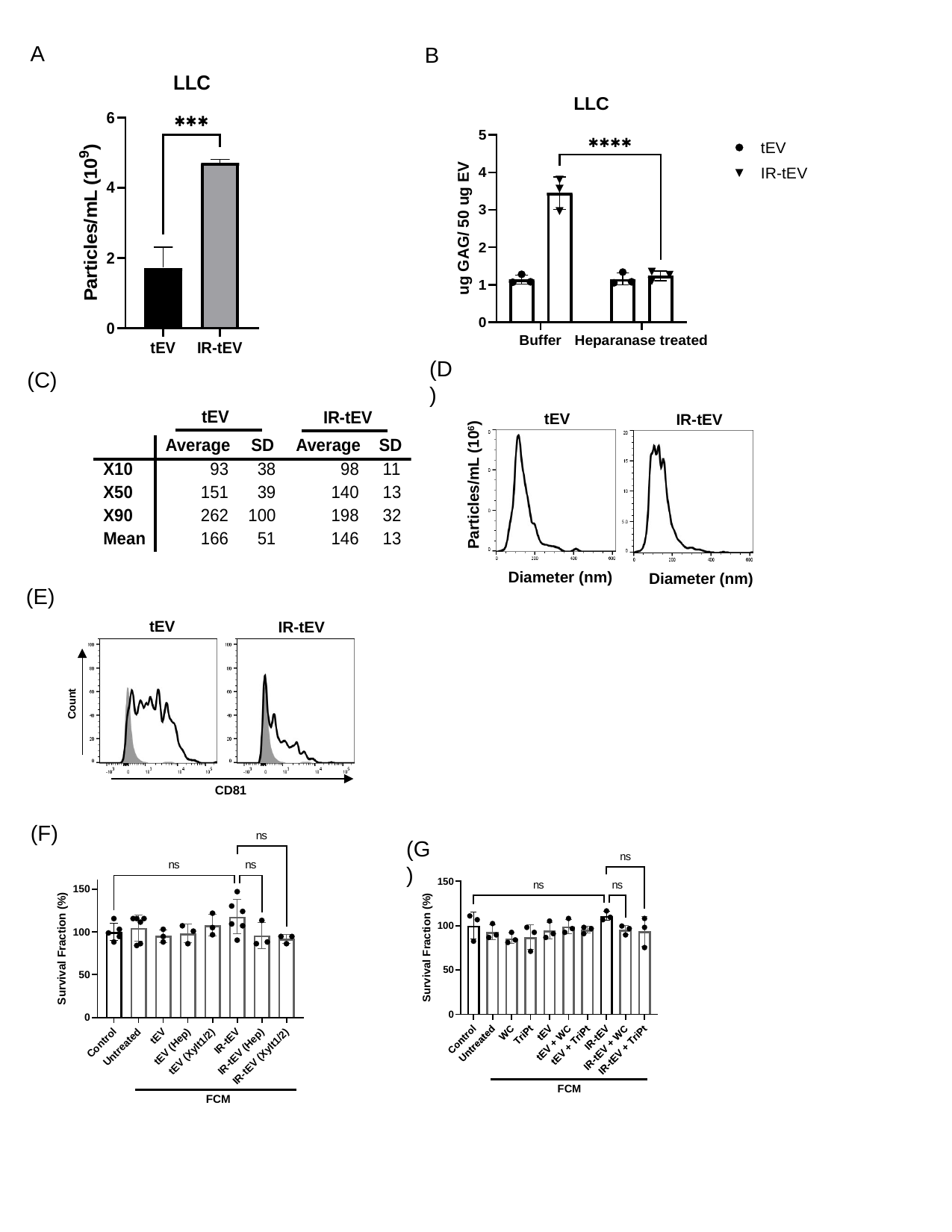

A
(C)
B
(D)
tEV
IR-tEV
Particles/mL (106)
Diameter (nm)
Diameter (nm)
(E)
tEV
IR-tEV
CD81
Count
(F)
(G)
